# Acute Gastric Necrosis in a Teenager

**DOI:** 10.1155/2020/8882179

**Published:** 2020-09-25

**Authors:** Joseph Yorke, Frank Enoch Gyamfi, Ronald Awoonor-Williams, Ebenezer Osei-Akoto, Emmanuel Acheampong, Emmanuella Nsenbah Acheampong, Michael Ofoe Adinku, Francis Akwaw Yamoah, Thomas Okpoti Konney, Ernest Adjei, Edward Amoah Boateng, Charles Kofi Dally, Kwabena Acheamfour Ababio, Dennis Afful-Yorke, Dorcas Ahulu

**Affiliations:** ^1^General Surgery Unit, Directorate of Surgery, Komfo Anokye Teaching Hospital (KATH), Kumasi, Ghana; ^2^Department of Surgery, School of Medicine and Dentistry, College of Health Sciences, Kwame Nkrumah University of Science and Technology, Kumasi, Ghana; ^3^Department of Molecular Medicine, School of Medicine and Dentistry, Kwame Nkrumah University of Science and Technology, Kumasi, Ghana; ^4^Directorate of Obstetrics and Gynaecology, Komfo Anokye Teaching Hospital, Kumasi, Ghana; ^5^Directorate of Pathology, Komfo Anokye Teaching Hospital, Kumasi, Ghana; ^6^Directorate of Anaesthesia and Intensive Care, Komfo Anokye Teaching Hospital, Kumasi, Ghana

## Abstract

Gastric infarction is a rare condition often associated with high mortality due to a delay in diagnosis. The stomach which has a rich supply of blood is a rare site for such a condition. Gastric infarction has a long list of etiological factors. We report a case of a patient who was managed successfully following gastric infarction from gastric dilatation. An 18-year-old female student presented with a three-day history of abdominal pain associated with abdominal distension of two days. The abdomen was distended with generalized tenderness, rebound tenderness, and guarding. Bowel sounds were absent. Digital rectal examination was unremarkable, and a pregnancy test was negative. Biochemical tests were all normal. Intraoperatively, two litres of serosanguinous fluid was suctioned from the abdomen. About 300 mL of pus was suctioned from the pelvis. The gangrenous portion was resected, and repair was done in two layers using Conell and Lambert suture techniques. Acute gastric necrosis is a rare surgical condition that requires a high index of suspicion and prompts aggressive resuscitation and surgical intervention to obviate the high mortality rate associated with the condition.

## 1. Introduction

Acute gastric gangrene is rare due to the rich and anastomotic nature of blood supply to the stomach [1, 2]. Only a few cases of gastric gangrene have been reported in the literature. The first case of a major gastric infarct was reported in 1909 by Bauman [1]. The stomach is a well-vascularized organ with four major vascular supplies; hence, infarction of the stomach is an uncommon phenomenon compared to other parts of the gastrointestinal tract [3].

Several possible causes have been assigned to gastric gangrene. Notable among these is herniation of the stomach through the diaphragm, gastric volvulus, acute necrotizing gastritis, complications after abdominal surgery, anorexia nervosa and bulimia, trauma, exposure to caustic materials, diabetes mellitus, and acute massive gastric dilatation from binge eating or pathological aerophagia [4–10]. The condition may be confused with other causes of peritonitis. This is because of its rarity and thus mostly diagnosed intraoperatively [11, 12].

## 2. Case Report

An 18-year-old female student presented at the Accident and Emergency unit at the Komfo Anokye Teaching Hospital (KATH) with a three-day history of abdominal pain associated with abdominal distension of two days. The pain which was sudden in onset started four hours after a heavy evening meal. The pain was initially localized to the epigastrium. It had no known aggravating or relieving factors. The pain progressively worsened and became generalized the day after onset. It was associated with abdominal distension and absolute constipation. She was seen at a peripheral hospital and managed for a day with intravenous fluids, antibiotics, and subsequently referred for further management due to worsening of her condition. There was no history of fever, vomiting, had no history of peptic ulcer disease, and was not on NSAIDs or steroids. She had no history of alcohol use.

On examination, she was conscious and alert, not pale, anicteric, well hydrated, and had a temperature of 37.8°C. Her pulse rate was 110 beats per minute, regular, and good volume. Extremities were warm, and blood pressure was 130/80 mmHg. Heart sounds were normal with no murmurs. Respiratory rate was 22 cycles per minute. The chest was clinically clear, and SPO_2_ was 96% on room air.

The abdomen was distended with generalized tenderness, rebound tenderness, and guarding. Bowel sounds were absent. Digital rectal examination was unremarkable, and a pregnancy test was negative. Laboratory investigations revealed the following: Hb (11.0 g/dL), WBC (34.28 × 10^3^/UL), and neutrophils (90.8%). Biochemical tests were all normal (Table 1). A diagnosis of generalized peritonitis 2° to hollow viscous perforation was made. The patient was resuscitated with intravenous fluids, broad-spectrum antibiotics, and analgaesia. Nasogastric decompression as part of resuscitation yielded instantly two litres of brownish fluid with another 600 mL before surgical intervention. Informed consent was obtained from the patient and her parents and sent to the theatre for laparotomy.

**Figure 1:**
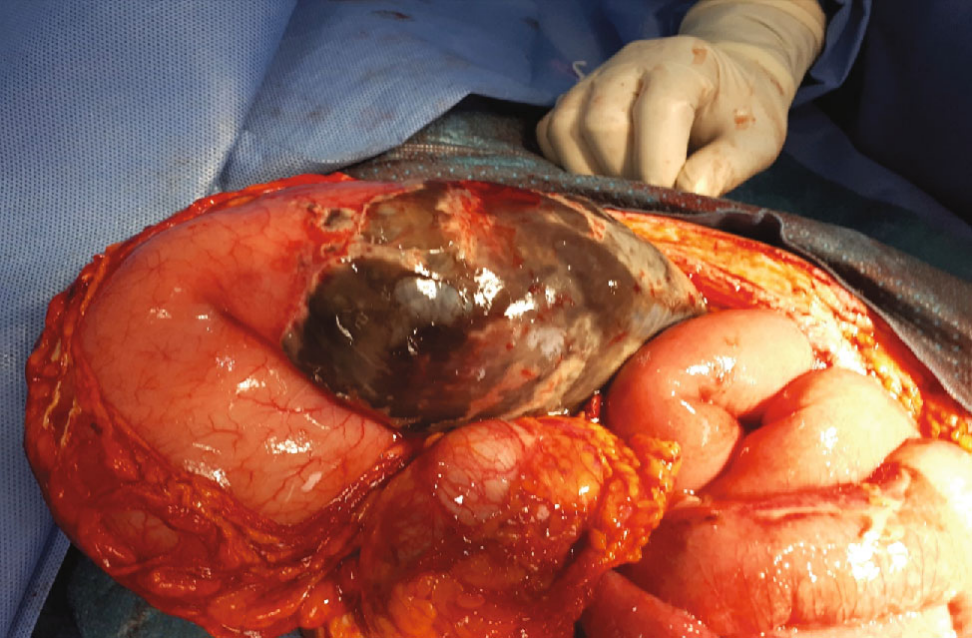
Appearance of the stomach after exposing the posterior wall.

**Figure 2:**
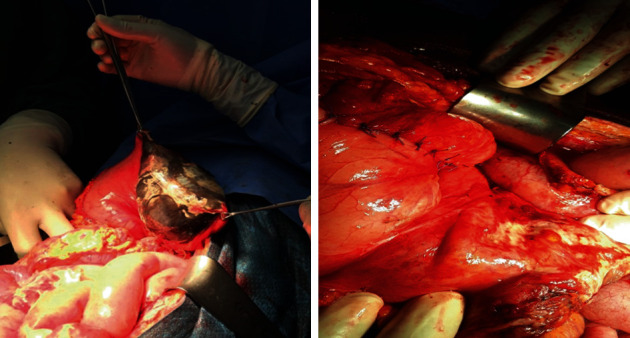
Excision of the necrotic portion of the stomach.

**Figure 3:**
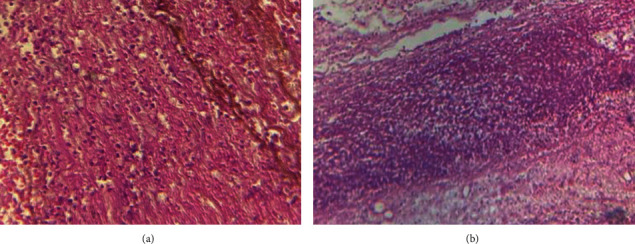
(a, b) Histopathological micrographs of partial gastrectomy tissues.

Intraoperatively, two litres of serosanguinous fluid was suctioned from the abdomen. About 300 mL of pus was suctioned from the pelvis. The stomach was grossly dilated and edematous with gangrene of the posterior wall about 18 × 12cm, 5 cm distal to the fundus. The vasculature of the stomach was intact. The rest of the gastrointestinal tract was normal. The gangrenous portion was resected, and repair was done in two layers using Conell and Lambert suture techniques (Figures 1 and 2). The partial gastrectomy specimen for histopathology looks gangrenous with patchy areas of fibrinoid exudates on gross sections which show dark brown appearance (Figure 3). The abdomen was lavaged with warm saline and closed up. Postoperative recovery was uneventful. She started receiving oral intake on postoperative day five.

Among the liver function parameters, aspartate transaminase (AST) levels were high (83.4 *μ*/L). There were low levels of albumin (25.4 g/L), and the bun to creatinine ratio was above the reference range (36.7) (Table 1).

Figures 1 and 2 show the appearance of the stomach after exposing the posterior and the excision of the necrotic portion of the stomach.

The section of gastric showed transmural necrosis with congested blood vessels. There was no atypia (Figure 3).

## 3. Discussion

Acute gastric necrosis is a rare occurrence and mostly diagnosed intraoperatively. Because of its rarity, diagnosis is mostly delayed and virtually made only at laparotomy [11, 12]. Findings from physical examination can differ widely from one patient to the other. Diagnosis can be supported by radiological imaging of gastric dilatation and pneumoperitoneum. Early diagnosis is significant, and CT scan is relevant in revealing diagnosis and etiology [13, 14]. The cardinal consequence of binge eating as occurred in the index case is acute gastric dilatation which has been implicated as the key pathway leading to gastric necrosis in more than half the cases reported [14, 15].

In the event of massive gastric dilatation, a resultant pressure of about 14 mmHg is sufficient enough to cause venous insufficiency leading to ischaemia [16]. Intragastric volumes of at least 3 litres have been found culpable in the generation of such pressure. In the index case, about 2.6 litres was drained before surgical intervention though short of the volumes noticed in experimental models. Perhaps such volumes may not necessarily be required for the generation of such pressure or there may be other factors accounting for it. Reported cases of gastric necrosis amidst acute gastric dilatation occurred in patients with eating disorders such as bulimia [10]. This patient had no history of any eating disorder.

Circulatory collapse has been a key feature of this condition in literature or several reported cases [3, 17, 18]. In our case, the circulatory collapse was not a key feature even with the long duration before surgical intervention. This could be attributed to the fact that the patient had intravenous infusion given at a peripheral hospital before coming to our centre. The management of acute gastric necrosis involves resection of the necrotic portion of the stomach with the establishment of gastrointestinal continuity or resorting to proximal diversion with a distal stoma in stable and unstable patients, respectively, as well as the extent of peritoneal soiling irrespective of patient stability [2, 3, 9, 19]. In the current case, the patient was clinically stable which allowed resection plus establish gastrointestinal continuity.

The mortality associated with acute gastric necrosis ranges from 50 to 80 percent which underscores the severity or life-threatening nature of the condition [19]. Significant mortalities have been reported in recent publications even with aggressive management [3, 16, 19]. The presence of circulatory collapse may be a significant contribution to mortality. In our case, the patient had been seen at a peripheral hospital where some fluid correction had been instituted. This may account for the different picture in terms of outcome.

## 4. Conclusion

Acute gastric necrosis is a rare surgical condition that requires a high index of suspicion and prompts aggressive resuscitation and surgical intervention to obviate the high mortality rate associated with the condition.

## Figures and Tables

**Table 1:** Patient's biochemical test.

Test	Result	Unit	Reference range
Liver function test			
AST	83.4	*μ*/L	0.0-32.0
ALT	29.1	*μ*/L	0.0-33.0
ALP	33.8	*μ*/L	26.0-129.0
GGT	36.5	*μ*/L	6.0-42.0
Total protein	45.9	g/L	66.0-87.0
Albumin	25.4	g/L	35.0-82.0
Globulin	20.5	g/L	25.0-35.0
Bilirubin, total	4.4	*μ*mol/L	0.0-17.0
Bilirubin, direct	0.8	*μ*mol/L	0.0-3.4
Bilirubin, indirect	3.6	*μ*mol/L	1.5-14.0
Renal function test			
Urea	2.15	mmol/L	2.50-8.30
Creatine	27	*μ*mol/L	44-90
Bun to creatinine ratio	36.7		8.0-350

AST: aspartate transaminase; ALT: alanine transaminase; ALP: alkaline transaminase; GGT: gamma glutamyl transaminase.

## Data Availability

The data used and/or analysed during the current study are within the manuscript.
